# Cytoplasmic deadenylase Ccr4 is required for translational repression of *LRG1* mRNA in the stationary phase

**DOI:** 10.1371/journal.pone.0172476

**Published:** 2017-02-23

**Authors:** Duong Long Duy, Yasuyuki Suda, Kenji Irie

**Affiliations:** 1 Department of Molecular Cell Biology, Graduate School of Comprehensive Human Sciences and Faculty of Medicine, University of Tsukuba, Tsukuba, Japan; 2 Live Cell Super-resolution Imaging Research Team, RIKEN Center for Advanced Photonics, Wako, Saitama, Japan; John Curtin School of Medical Research, AUSTRALIA

## Abstract

Ccr4 is a major cytoplasmic deadenylase involved in mRNA poly(A) tail shortening in *Saccharomyces cerevisiae*. We have previously shown that Ccr4 negatively regulates expression of *LRG1* mRNA encoding a GTPase-activating protein for the small GTPase Rho1, a component of cell wall integrity pathway, and deletion of *LRG1* suppresses the temperature-sensitive growth defect of the *ccr4Δ* mutant. We have also shown that the slow growth of the *ccr4Δ* mutant is suppressed by deletion of another gene, *PBP1*, encoding a poly(A)-binding protein (Pab1)-binding protein 1; however, the underlying mechanism still remains unknown. In this study, we investigated how *ccr4Δ* and *pbp1Δ* mutations influence on the length of poly(A) tail and *LRG1* mRNA and protein levels during long-term cultivation. In the log-phase *ccr4Δ* mutant cells, *LRG1* poly(A) tail was longer and *LRG1* mRNA level was higher than those in the log-phase wild-type (WT) cells. Unexpectedly, Lrg1 protein level in the *ccr4Δ* mutant cells was comparable with that in WT. In the stationary-phase *ccr4Δ* mutant cells, *LRG1* poly(A) tail length was still longer and *LRG1* mRNA level was still higher than those in WT cells. In contrast to the log phase, Lrg1 protein level in the stationary-phase *ccr4Δ* mutant cells was maintained much higher than that in the stationary-phase WT cells. Consistently, active translating ribosomes still remained abundant in the stationary-phase *ccr4Δ* mutant cells, whereas they were strongly decreased in the stationary-phase WT cells. Loss of *PBP1* reduced the *LRG1* poly(A) tail length as well as *LRG1* mRNA and protein levels in the stationary-phase *ccr4Δ* mutant cells. Our results suggest that Ccr4 regulates not only *LRG1* mRNA level through poly(A) shortening but also the translation of *LRG1* mRNA, and that Pbp1 is involved in the Ccr4-mediated regulation of mRNA stability and translation.

## Introduction

In the nucleus of eukaryotic cells, mRNAs are transcribed and then undergo modifications including addition of the cap 7-methylguanosine (m7G) to the 5' end, addition of poly(A) tail to the 3' end, and splicing to remove introns [1]. The mRNAs are then transported to the cytoplasm, where the extensive regulation steps happen to control mRNAs fate, these processes are so-called post-transcriptional regulation. In the cytosol, the Pab1 (Poly[A] binding protein 1) binds to poly(A) tail of mRNAs and physically interacts with the translational initiation factor eIF4G, a component of the translational initiation complex. Another component of this complex, eIF4E, directly binds to the 5' cap structure of mRNA to form mRNP (messenger ribonucleoprotein) loop, which is dependent on 5' cap and 3’ poly(A) tail. The loop formation recruits ribosome subunits and other initiation factors to mRNAs to initiate translation [2–4]. In addition to translation, mRNA degradation also occurs simultaneously. mRNA degradation firstly initiates with shortening poly(A) tail by the cytoplasmic deadenylase [5, 6]. When the deadenylase accesses poly(A) tail, it trims the tail to a certain length to release Pab1 and disrupts the mRNP loop. The 5' cap structure is then removed by the Dcp1-Dcp2 decapping complex. The decapped 5' end is subjected to the 5'-3' degradation by the XrnI exonuclease, whereas the 3' end with truncated poly(A) tail is subjected to 3'-5' degradation by exosome [5, 6]. Regulation of mRNA poly(A) tail length is an important step that determines the mRNA behavior in the cell. RNA-binding proteins such as PUF (Pumilio and FBF) proteins or miRNAs, which bind to the specific sites in the 3'-untranslated region (UTR) of mRNAs, regulate mRNA degradation and/or translation through recruiting the mRNA decay machinery to the target mRNAs [7–9].

In *Saccharomyces cerevisiae*, the major cytoplasmic deadenylase is Ccr4 (Carbon catabolite repression 4), a catalytic component of Ccr4-Not complex. The Ccr4-Not multi-subunit complex is conserved from yeast to human, and plays a crucial role in gene expression regulation due to its deadenylation and ubiquitination functions [6, 10]. It is supposed that long poly(A) tail length would enhance mRNA stability and translation [6, 11]. However, it is reported that the protein levels of genes encoding septin and a regulator of septin assembly, such as *CDC11* and *CDC42*, are not increased in the *ccr4Δ* mutant, although these mRNAs have longer poly(A) tails in the *ccr4Δ* mutant than those in wild-type (WT) cells [12]. The *ccr4Δ* mutant shows pleiotropic phenotypes including cell checkpoint defect, aberrant septin organization, weak cell lysis, and cell growth defect. The multiple defects may be caused by the aberrant expression of the target mRNAs of Ccr4, and each of phenotypes can be suppressed by deletion of the related specific genes [12–16].

The growth defect of the *ccr4Δ* mutant can be suppressed by deletion of *PBP1* (Pab1 binding protein 1) [14, 15]. Pbp1 is an yeast ortholog of human ataxin-2, which is thought to associate with neurodegenerative diseases [17]. Pbp1 together with Mkt1 is reported to regulate the translation of *HO* mRNA [18]. Pbp1 is also reported to associate with translating ribosomes and to be present in the stress granule [18, 19]. Pbp1 is supposed to negatively regulate the Pan2-Pan3 complex, another cytoplasmic deadenylase, which contributes to regulation of mRNA poly(A) tail length [20, 21]. Phosphorylation of Pbp1 inhibits TORC1 (target of rapamycin complex 1) by separating it to the stress granule to control cellular growth and proliferation [22]. Since loss of *PBP1* has no obvious phenotype in normal growth condition, the cellular function of Pbp1 still remains unclear.

Previously, we have shown that Ccr4 negatively regulates expression of *LRG1* mRNA encoding for a GTPase activating protein (GAP) which inactivates the small GTPase Rho1 involved in the cell wall integrity (CWI) pathway [13]. Deletion of *CCR4* perturbs the regulation of the CWI pathway lead to cell lysis and temperature-sensitive growth defect. Loss of *LRG1* can suppress the temperature-sensitive growth defect of the *ccr4Δ* mutant [13, 23]. Since loss of *PBP1* also suppresses the growth defect of the *ccr4Δ* mutant [14, 15], we thought that Pbp1 may be involved in the regulation of the *LRG1* expression. The *LRG1* mRNA would be a potential candidate to study the role of Ccr4 and Pbp1 in the post-transcriptional regulation.

In this study, we investigated the *LRG1* poly(A) tail length and the *LRG1* mRNA and protein levels in both the log and stationary phases. The *LRG1* mRNA in the *ccr4Δ* mutant harbored longer poly(A) tail than that in WT cells in both the log and stationary phases, however, Lrg1 protein was up-regulated only in the stationary phase but not in the log phase. Polysome analysis revealed that the abundant active translating ribosomes still exist in the stationary-phase *ccr4Δ* mutant cells, while they were strongly decreased in the stationary-phase WT cells. Deletion of *PBP1* reduced *LRG1* poly(A) tail length and *LRG1* mRNA and protein levels in the stationary-phase *ccr4Δ* mutant cells, but it did not suppress the abundant polysomes. We also found that an RNA-binding protein Puf5/Mpt5 is involved in the regulation of *LRG1* expression in the stationary phase. The other targets of Puf5, including *MCM2*, *MCM4*, *MCM7*, and *ELM1*, also showed the expression pattern similar to that of *LRG1*. Our data suggest that Ccr4 is required for the translational repression of Puf5-target mRNAs and the global translational repression in the stationary phase. Our data also suggest that Pbp1 is involved in the Ccr4-mediated regulation of mRNA stability and translation.

## Results

### Ccr4 negatively regulates poly(A) tail length and level of *LRG1* mRNA

**Fig 1:**
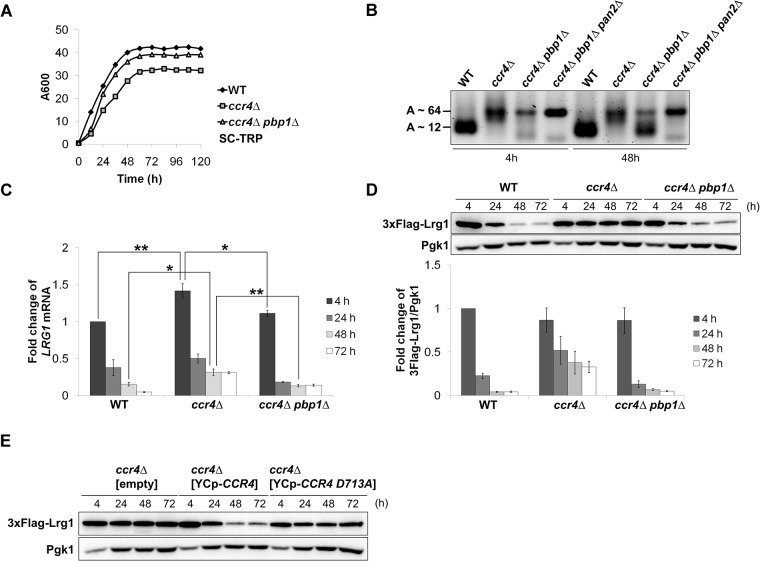
*LRG1* mRNA and protein levels were increased in the stationary-phase *ccr4Δ* mutant. (A) The growth curves of WT, *ccr4Δ*, and *ccr4Δ pbp1Δ* cells in SC-Trp media. The strains harboring the plasmid pRS314-3FLAG-LRG1 were pre-cultured overnight and then transferred into fresh SC-Trp media to grow for 5 days at 28°C. The cell cultures were taken at the indicated times to measure A600 nm. (B) The *LRG1* poly(A) tail lengths in WT, *ccr4Δ*, *ccr4Δ pbp1Δ*, and *ccr4Δ pbp1Δ pan2Δ* mutant cells in the log phase (4 h) and the stationary phase (48 h). The strains were grown in YPD media from the log phase to the stationary phase at 28°C. The cells were collected at indicated time points for RNA isolation. The *LRG1* poly(A) tail was amplified using the poly(A) tail length kit. The average poly(A) tail lengths were determined by sequencing. (C) Expression of *LRG1* mRNA in WT, *ccr4Δ*, and *ccr4Δ pbp1Δ* mutants. The strains harboring the plasmid pRS314-3FLAG-LRG1 were grown at 28°C from the log phase to the stationary phase in SC-Trp media. The cells were collected at the indicated times for RNA isolation. The *LRG1* mRNA levels were quantified by qRT-PCR analysis, and the relative mRNA levels were calculated using delta delta Ct method normalized to *SCR1* reference gene. The data show mean ± SEM (n = 4) of fold change of *LRG1* mRNA from WT cells at 4 h of culture. *P < 0.05, **P < 0.01 as determined by Tukey’s test. (D) Expression of Lrg1 protein in WT, *ccr4Δ*, and *ccr4Δ pbp1Δ* mutants. The strains harboring the plasmid pRS314-3FLAG-LRG1 were grown at 28°C from the log phase to the stationary phase in SC-Trp media. The cells were collected at the indicated times, and cell extracts were prepared for immunoblotting with anti-Flag (3xFlag-Lrg1) and anti-Pgk1 antibodies. The intensities of 3xFlag-Lrg1 signals were measured and normalized to the Pgk1 signals. The values are plotted as the fold change from WT cells at 4 h of culture. The data show mean ± SEM (n = 3). (E) The deadenylase activity of Ccr4 is required for the regulation of *LRG1* expression. The plasmid YCplac33-CCR4 or plasmid YCplac33-CCR4-D713A or empty vector was transformed into the *ccr4Δ* mutant cells harboring plasmid pRS314-3FLAG-LRG1. Transformants were grown at 28°C from the log phase to the stationary phase in SC-Trp-Ura media. The cells were collected at the indicated times, and cell extracts were prepared for immunoblotting with anti-Flag (3xFlag-Lrg1) and anti-Pgk1 antibodies. Pgk1 was used as the loading control.

Traven et al. have shown that, in the *ccr4Δ* mutant, mRNAs encoding septin and regulators of septin assembly, such as *SHS1*, *CDC11*, *CDC42*, *CDC24*, *RGA1*, and *ELM1*, harbor longer poly(A) tail; however, the levels of these mRNAs are not increased. Within them, Cdc11 and Cdc42 protein levels appear not to be increased [12]. We examined poly(A) tail length, *LRG1* mRNA level, and Lrg1 protein level in WT and *ccr4Δ* mutant harboring the FLAG-LRG1 plasmid. This FLAG-LRG1 plasmid contains endogenous *LRG1* promoter, the coding sequence of *LRG1* gene fused with 3xFLAG tag at N terminal, and *LRG1* 3'-UTR. In this experiment, we cultured the cells in longer time, up to 120 h. The WT and *ccr4Δ* mutant reached saturated cell density after 60 h of culture (Fig 1A). We harvested the cells at the time points including 4 h, 24 h and 48 h, and 72 h, corresponding to the early log phase, the late log phase, and the stationary phase, respectively. In agreement with Traven’s report [12], the *LRG1* poly(A) tail lengths in the *ccr4Δ* mutant were longer than those in WT (Fig 1B, WT vs *ccr4Δ*, 4 h and 48 h). Consistent with the fact that poly(A) tail length is important for mRNA stability, *LRG1* mRNA levels in the *ccr4Δ* mutant were higher than those in WT through the time course (Fig 1C, 4 h, 24 h, 48 h, 72 h). In WT cells, the *LRG1* mRNA level dramatically dropped throughout the time course (Fig 1C, WT). In contrast, in *ccr4Δ* mutant, the *LRG1* mRNA level initially dropped during 4 h to 24 h time points, but it minimally changed and remained relatively high level after the 24 h time point (Fig 1C, *ccr4Δ*). These results suggest that Ccr4 negatively regulates the poly(A) tail length and the *LRG1* mRNA level, and that the longer poly(A) tail seems to be more important for the mRNA level at the later time points of cell growth.

### Lrg1 protein level is up-regulated in the stationary-phase *ccr4Δ* mutant cells

We then examined the Lrg1 protein levels in WT and *ccr4Δ* mutant (Fig 1D). At the 4 h time point, Lrg1 protein level in *ccr4Δ* mutant was similar to that in WT, although the *LRG1* mRNA level in *ccr4Δ* mutant was slightly higher than that in WT (Fig 1C and 1D, WT vs *ccr4Δ*, 4 h). This data also suggests that the longer poly(A) tail of *LRG1* mRNA has little effect on Lrg1 protein level at this 4 h time point. Correlated with the observation that the *LRG1* mRNA level in WT dramatically dropped throughout the time course (Fig 1C, WT), the Lrg1 protein level in WT also dramatically dropped throughout the time course (Fig 1D, WT). In the *ccr4Δ* mutant, as the *LRG1* mRNA remained relatively high level even at the 24 h, 48 h, and 72 h time points (Fig 1C, *ccr4Δ*), Lrg1 protein levels also remained relatively high level even at 24 h the 48 h and 72 h time points (Fig 1D, *ccr4Δ*). The Lrg1 protein levels in the *ccr4*Δ mutant were continuously maintained higher than those in WT up to 120 h of the culture (data not shown). It is noted that, at 48 h time point, the *LRG1* mRNA level in *ccr4Δ* mutant was 2-fold higher than that in WT (Fig 1C, WT vs *ccr4Δ*, 48 h), but Lrg1 protein level in *ccr4Δ* mutant was 8.9-fold higher than that in WT (Fig 1D, WT vs *ccr4Δ*, 48 h). The relative Lrg1 protein level/ *LRG1* mRNA level ratios in WT and *ccr4Δ* mutant cells at this 48 h time point were 0.276 and 1.196, respectively. Thus, the effect of *ccr4Δ* mutation on Lrg1 protein level was dominant compared to that on *LRG1* mRNA level at the 48 h time point. In addition, the *LRG1* poly(A) tail length in the *ccr4Δ* mutant was also longer than that in WT at 48 h of the cultures (Fig 1B, lane 5, 6). These data suggest that Ccr4 negatively regulates not only the *LRG1* mRNA level through the poly(A) shortening, but also the translation efficiency of *LRG1* mRNA.

To assess the role of the deadenylase activity of Ccr4 in the regulation of *LRG1* expression, the catalytic residue of Ccr4, Asp-713, which is required for *in vitro* deadenylase activity, was mutated to alanine [24]. While the wild-type *CCR4* gene could decrease the high Lrg1 protein level in the stationary-phase *ccr4Δ* mutant cell, the *CCR4-D713A* gene could not (Fig 1E). Consistently, the wild-type *CCR4* gene, but not *CCR4-D713A*, complemented the growth defect of *ccr4Δ* mutant (data not shown). Thus, the deadenylase activity of Ccr4 is required for the regulation of *LRG1* expression.

### Active translating polysomes are abundant in the stationary-phase *ccr4Δ* mutant cells

**Fig 2:**
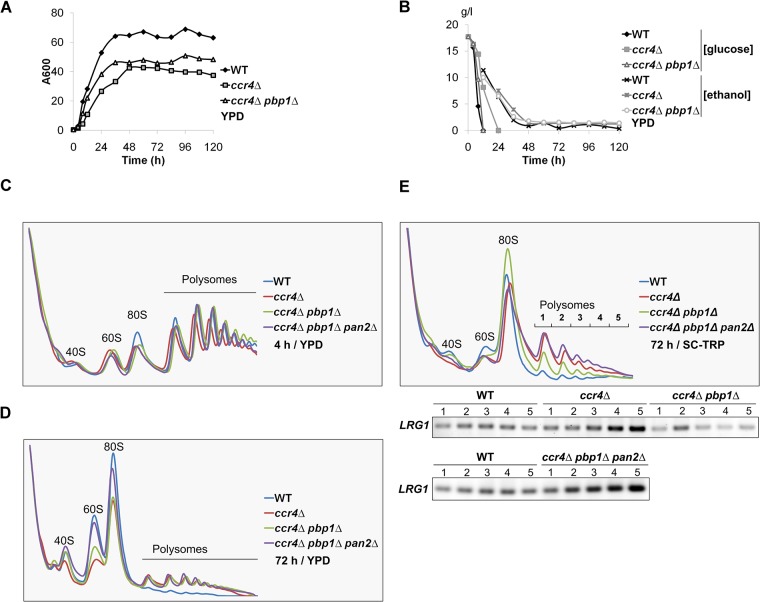
Active translating polysomes were still abundant in the stationary-phase *ccr4Δ* mutant. (A) Growth curves of WT, *ccr4Δ*, and *ccr4Δ pbp1Δ* cells in YPD media. The strains were pre-cultured overnight and then transferred into fresh YPD media to grow for 5 days at 28°C. The cell cultures were taken at the indicated times to measure A600 nm. (B) The WT, *ccr4Δ*, and *ccr4Δ pbp1Δ* mutant cells went into the stationary phase after 48 h of culture in YPD media. The strains were pre-cultured overnight in YPD media and then transferred into fresh YPD media to grow for 5 days at 28°C. The cultures were taken at the indicated times to measure glucose concentration. The ethanol concentrations were measured after glucose in the media had been depleted. (C) Polysome analyses of WT, *ccr4Δ*, *ccr4Δ pbp1Δ*, and *ccr4Δ pbp1Δ pan2Δ* mutant cells in the log phase (4 h). The strains were pre-cultured overnight in YPD media and then transferred into fresh YPD media to grow for 4 h at 28°C. The cells were collected and cell lysates were prepared for polysome analysis as described in material and methods. (D) Polysome analyses of WT, *ccr4Δ*, *ccr4Δ pbp1Δ*, and *ccr4Δ pbp1Δ pan2Δ* mutant cells in the stationary phase (72 h) in YPD. The strains were pre-cultured overnight in YPD media and then transferred into fresh media to grow for 72 h at 28°C. The cells were collected and cell lysates were prepared for polysome analysis as described in material and methods. (E) Polysome analyses and *LRG1* mRNA levels of WT, *ccr4Δ*, *ccr4Δ pbp1Δ*, and *ccr4Δ pbp1Δ pan2Δ* mutant cells in the stationary phase (72 h) in SC-Trp media. The strains were pre-cultured overnight in SC-Trp media and then transferred into fresh media to grow for 72 h at 28°C. The cells were collected and cell lysates were prepared for polysome analysis as described in material and methods. The same volumes of RNA isolated from each of polysome fractions were subjected to RT-PCR to synthesize cDNAs. The *LRG1* cDNA was amplified using Taq polymerase. The data show the relative amounts of *LRG1* cDNA from the polysome fractions of the strains. We obtained similar results in two independent experiments and show a representative.

The Ccr4 deadenylase has been shown to associate with polysomes [25] and the Ccr4 ortholog in *Xenopus laevis* oocytes has been shown to have translational repression activity [26]. We therefore examined whether Ccr4 negatively regulates the translation in the later growth phase (i.e. 48 h or later time point in Fig 1). In this time, we cultured the cells not harboring the FLAG-LRG1 plasmid in YPD media (Fig 2A), and determined the exact growth phases based on the glucose and ethanol levels [27]. The WT cells used up glucose and went into the post diauxic-shift after 12 h of culture, whereas the *ccr4Δ* mutant cells took 24 h (Fig 2B). After glucose was exhausted in the media, the cells turned to utilize ethanol and went into the stationary phase after 48 h of culture, when the cell densities were saturated (Fig 2A) and ethanol was depleted in the media (Fig 2B). We then performed polysome analysis of WT and *ccr4Δ* mutant at 4 h and 72 h of culture corresponding to the log phase and the stationary phase, respectively. Polysome profiles revealed that translation was active in both WT and *ccr4Δ* mutant at the 4 h time point when the carbon source was abundant (Fig 2C). In this stage, the active translating polysomes were dominant compared with ribosome 80S, 60S, and 40S (Fig 2C). It has been reported that, in the stationary phase when the carbon source is depleted, WT cells strongly reduce the protein synthesis and many other metabolic processes [27]. Consistently, the active polysomes were strongly decreased in WT cells at 72 h time point (Fig 2D). In contrast, in the *ccr4Δ* mutant, the active polysomes were also decreased, but still remained more abundant than that in WT cells at 72 h time point (Fig 2D). We obtained essentially the same data using SC-Trp media (Fig 2E). Although, in the culture using SC-Trp media, the active polysomes remained at low level in WT, the active polysomes remained much more abundant in the *ccr4Δ* mutant. These results indicate that Ccr4 indeed negatively regulates the translation in addition to the mRNA level. The active polysomes remained abundant even in the stationary phase, suggesting that Ccr4 seems to be required for global translational repression in the stationary phase rather than the translation of specific mRNA, *LRG1* mRNA.

To confirm whether translation of the *LRG1* mRNA was increased in the stationary-phase *ccr4Δ* mutant cells, we then examined the *LRG1* mRNA level in each polysome fractions from WT and *ccr4Δ* mutant cells at 72 h of culture. The same volumes of purified mRNAs from each polysome fraction were subjected to RT-PCR reactions to generate cDNAs used as the template for *LRG1* amplification. As predicted, *LRG1* mRNA was more enriched in heavy polysome fractions in the *ccr4Δ* mutant than those in WT (Fig 2E). This result reveals that the translation of *LRG1* mRNA was increased in the stationary-phase *ccr4Δ* mutant cells, which lead to the increase in Lrg1 protein levels (Fig 1D).

### Loss of *PBP1* reduces Lrg1 level in the stationary-phase *ccr4Δ* mutant cells

We have previously reported that deletion of *PBP1* suppressed the slow growth defect and temperature-sensitive growth defect of the *ccr4Δ* single and the *ccr4Δ khd1Δ* double mutants [14]. We also found that the *pbp1Δ* mutation did not suppress the increased *LRG1* mRNA level of the *ccr4Δ khd1Δ* mutant [14]. Since, in our previous experiment, we had measured the *LRG1* mRNA and protein levels only in the log phase culture, we re-examined the *LRG1* mRNA and protein levels in WT, *ccr4Δ*, and *ccr4Δ pbp1Δ* mutants in the longer time course (Figs 1A and 2A). As shown in Figs 1A and 2A, the *ccr4Δ pbp1Δ* mutant showed better growth than the *ccr4Δ* mutant in both SC-Trp and YPD media. The *ccr4Δ pbp1Δ* mutant reached the stationary phase after 60 h of culture in SC-Trp media (Fig 1A) and 48 h of culture in YPD media (Fig 2A and 2B).

Then we examined the poly(A) tail length of *LRG1* mRNA, *LRG1* mRNA level, and Lrg1 protein level in the *ccr4Δ pbp1Δ* mutant harboring the FLAG-LRG1 plasmid. It has been reported that Pbp1 is involved in the regulation of poly(A) tail length [21]. In addition, the cell extract of the *pbp1Δ* mutant in the stationary phase has shown stronger deadenylase activity *in vitro* compared to that in the log phase [20]. At the 4 h time point, *LRG1* poly(A) tail length in the *ccr4Δ pbp1Δ* mutant as well as that in the *ccr4Δ* mutant was longer than that in WT (Fig 1B, lanes 1, 2, 3). However, at the 48 h time point, a large portion of the *LRG1* mRNAs in the *ccr4Δ pbp1Δ* mutant harbored shorter poly(A) tail than those in the *ccr4Δ* mutant (Fig 1B, lane 6, 7). The *LRG1* mRNA levels in the *ccr4Δ pbp1Δ* mutant were decreased compared to those in the *ccr4Δ* mutant throughout the time course (Fig 1C, *ccr4Δ* and *ccr4Δ pbp1Δ*). Interestingly, although the Lrg1 protein levels in the *ccr4Δ pbp1Δ* mutant were also decreased compared to those in the *ccr4Δ* mutant throughout the time course (Fig 1D), the decrease in Lrg1 protein level was more evident than the decrease in the mRNA level. While the *LRG1* mRNA levels in the *ccr4Δ pbp1Δ* mutant was 2-fold lower than those in the *ccr4Δ* mutant at 48 h and 72 h time points (Fig 1C), the Lrg1 protein levels in the *ccr4Δ pbp1Δ* mutant were decreased 5.7-fold and 6.7-fold compared to those in the *ccr4Δ* mutant at 48 h and 72 h time points, respectively (Fig 1D). These data suggest that the *pbp1Δ* mutation not only down-regulates the increased *LRG1* mRNA level but also abandons the translation of *LRG1* in the *ccr4Δ* mutant. Since the *ccr4Δ pbp1Δ* mutant had the shorter poly(A) tail of the *LRG1* mRNA than that in the *ccr4Δ* mutant at the 48 h time point (Fig 1B, lane 6, 7), the decrease in *LRG1* poly(A) tail length may account for the reduction of Lrg1 protein level in the *ccr4Δ pbp1Δ* mutant (Fig 1D). It should be noted that the Lrg1 protein levels in WT, *ccr4Δ*, and *ccr4Δ pbp1Δ* mutants were similar at the 4 h time point (Fig 1C and 1D), and that the effects on Lrg1 protein levels by the *ccr4Δ* and *pbp1Δ* mutations were found in the later growth phase such as 48 h and 72 h time points. We also examined the Lrg1 protein level in the stationary-phase *pbp1Δ* single mutant, but we could not find any difference compared to that in WT (data not shown), suggesting that the *pbp1Δ* mutation may only affect the translation of the mRNAs harboring longer poly(A) tail in the *ccr4Δ* mutant.

### Deletion of *PBP1* does not reduce aberrant active polysomes in the stationary-phase *ccr4Δ* mutant cells

Because the *pbp1Δ* mutation reduced *LRG1* poly(A) tail length, *LRG1* mRNA level, and Lrg1 protein level in the *ccr4Δ* mutant in the later growth phase, we performed polysome analysis of *ccr4Δ pbp1Δ* mutant (Fig 2C,2D and 2E). Polysome profiles revealed that translation was similarly active in WT, *ccr4Δ*, and *ccr4Δ pbp1Δ* mutant at the 4 h time point (Fig 2C). Surprisingly, although the Lrg1 protein level in the *ccr4Δ pbp1Δ* mutant was much lower than that in the *ccr4Δ* mutant at the 72 h time point (Fig 1D), the active polysomes still remained abundant in the *ccr4Δ pbp1Δ* mutant similar to that in the *ccr4Δ* mutant (Fig 2D). The active polysomes also remained more abundant in both *ccr4Δ* and *ccr4Δ pbp1Δ* mutants than in WT at the 72 h time point in SC-Trp media (Fig 2E). These results indicate that deletion of *PBP1* does not reduce aberrant active polysomes in the stationary-phase *ccr4Δ* mutant, although the *pbp1Δ* mutation affects the translation of the *LRG1* mRNA.

Since the Lrg1 protein level in the *ccr4Δ pbp1Δ* mutant was much lower than that in the *ccr4Δ* mutant at the 72 h time point (Fig 1D), we next examined the *LRG1* mRNA level in each of polysome fractions from the *ccr4Δ pbp1Δ* mutant at 72 h time point (Fig 2E). Consistent with the decrease in Lrg1 protein level in the *ccr4Δ pbp1Δ* mutant at 72 h time point, *LRG1* mRNA was less enriched at heavy polysome fractions in the *ccr4Δ pbp1Δ* mutant than those in the *ccr4Δ* mutant (Fig 2E). Thus, Pbp1 may promote the association of *LRG1* mRNA to polysomes to enhance the translation in the absence of Ccr4.

### Regulation of *LRG1* expression by Ccr4 and Pbp1 is important for proper cell growth

**Fig 3:**
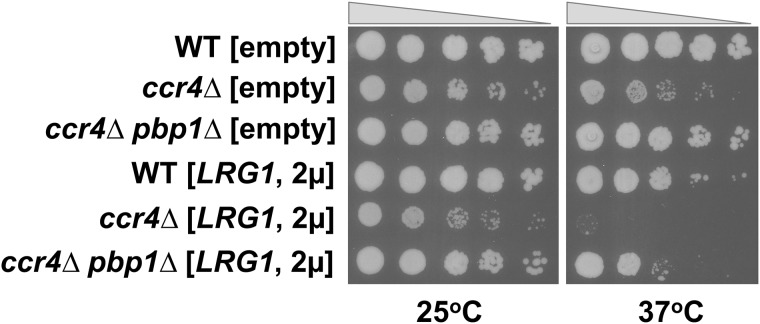
Overexpression of *LRG1* was toxic to the *ccr4Δ* mutant but not to the *ccr4Δ pbp1Δ* mutant at high temperature. The WT, *ccr4Δ*, and *ccr4Δ pbp1Δ* mutant strains harboring the plasmid YEplac195-LRG1 were grown at 28°C to the mid log phase. The same optical densities of cells were spotted onto SC-Ura plates and then incubated at 25°C or 37°C for 3 days.

The *LRG1* gene encoding for a GAP protein of the small GTPase Rho1, the key regulator of the CWI pathway, and high level of Lrg1 protein inhibits the cell growth at high temperature. To confirm whether the regulation of Lrg1 protein expression by Ccr4 and Pbp1 is important for growth control, we transformed a multi-copy plasmid carrying *LRG1* gene into WT, *ccr4Δ*, and *ccr4Δ pbp1Δ* mutant cells. As shown in Fig 3, overexpression of *LRG1* is more toxic to the *ccr4Δ* mutant, but less toxic to WT and *ccr4Δ pbp1Δ* mutants at 37°C. These data are consistent with that the increased Lrg1 protein level in the stationary-phase *ccr4Δ* mutant contributed to its slow growth, and that the decreased Lrg1 protein level by the *pbp1Δ* mutation also contributed to the suppression of the slow growth of the *ccr4Δ* mutant. Thus, Ccr4 and Pbp1 regulate the expression of *LRG1* gene together, and this regulation is important for proper cell growth.

### Pbp1 negatively regulates Pan2 activity in the absence of Ccr4 in the stationary phase

Mangus et al. have reported that Pbp1 negatively regulates mRNA poly(A) tail length through negative regulation of the Pan2 deadenylase activity [21]. We also reported that suppression of the *ccr4Δ* mutation by the *pbp1Δ* mutation is partly dependent on *PAN2* [14]. If Pan2 activity is inhibited by Pbp1, the *LRG1* poly(A) tail length in the *ccr4Δ pbp1Δ pan2Δ* triple mutant would be longer than that in the *ccr4Δ pbp1Δ* double mutant in the stationary phase. As predicted, whereas the poly(A) tail length of *LRG1* mRNA in the *ccr4Δ pbp1Δ* double mutant was decreased at the 48 h time point than that in the *ccr4Δ* mutant, the poly(A) tail length of *LRG1* mRNA in the *ccr4Δ pbp1Δ pan2Δ* mutant was not decreased (Fig 1B). The poly(A) tail length of *LRG1* mRNA in the *ccr4Δ pbp1Δ pan2Δ* mutant was around 64 bases that was similar to those in the *ccr4Δ* mutant (Fig 1B). These data suggest that the shortening of poly(A) tail length by Pbp1 is dependent on Pan2 activity in the stationary-phase *ccr4Δ* mutant.

**Fig 4:**
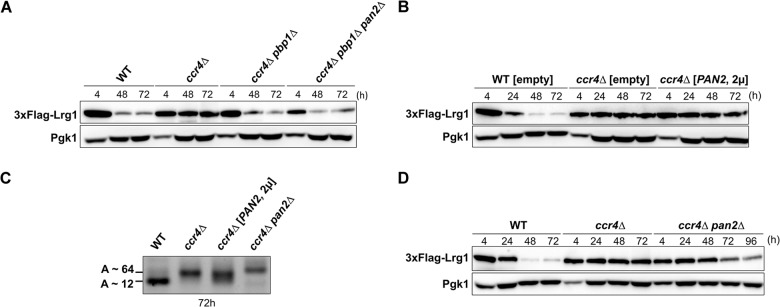
Effects of *PAN2* deletion, and *PAN2* overexpression on the expression of Lrg1 protein. (A) Effect of *PAN2* deletion on the expression of Lrg1 protein in *ccr4Δ pbp1Δ* mutant. The WT, *ccr4Δ*, *ccr4Δ pbp1Δ*, and *ccr4Δ pbp1Δ pan2Δ* mutant strains harboring the plasmid pRS314-3FLAG-LRG1 were grown at 28°C from the log phase to the stationary phase in SC-Trp media. The cells were collected at the indicated times, and cell extracts were prepared for immunoblotting with anti-Flag (3xFlag-Lrg1) and anti-Pgk1 antibodies. Pgk1 was used as the loading control. (B) Effect of *PAN2* overexpression on the expression of Lrg1 protein. The multi-copy plasmid YEplac195-PAN2 or empty vector was transformed into WT and *ccr4Δ* mutant cells harboring plasmid pRS314-3FLAG-LRG1. Transformants were grown at 28°C from exponential phase to the stationary phase in SC-Trp-Ura media. The cells were collected at the indicated times, and cell extracts were prepared for immunoblotting with anti-Flag (3xFlag-Lrg1) and anti-Pgk1 antibodies. Pgk1 was used as the loading control. (C) Effect of *PAN2* deletion and *PAN2* overexpression on *LRG1* poly(A) tail length in the stationary-phase *ccr4Δ* mutant cells. The strains were grown in SC-Trp-Ura media from the log phase to the stationary phase at 28°C. The cells were collected at 72 h time point for RNA isolation. The *LRG1* poly(A) tail was amplified using the poly(A) tail length kit. (D) Effect of *ccr4Δ pan2Δ* mutation on the expression of Lrg1 protein. The WT, *ccr4Δ*, and *ccr4Δ pan2Δ* mutant strains harboring plasmid pRS314-3FLAG-LRG1 were grown at 28°C from the log phase to the stationary phase in SC-Trp media. The cells were collected at the indicated times, and cell extracts were prepared for immunoblotting with anti-Flag (3xFlag-Lrg1) and anti-Pgk1 antibodies. Pgk1 was used as the loading control.

Then we examined the Lrg1 protein level in the *ccr4Δ pbp1Δ pan2Δ* mutant. Unexpectedly, the increase in *LRG1* poly(A) tail length did not result in the increase in Lrg1 level in the *ccr4Δ pbp1Δ pan2Δ* mutant in the stationary phase (Fig 4A). Thus, the translation of *LRG1* mRNA seems to require Pbp1 even in the absence of Pan2. We then performed polysome analysis of the *ccr4Δ pbp1Δ pan2Δ* mutant and found that the active polysomes still remained abundant in the *ccr4Δ pbp1Δ pan2Δ* mutant similar to that in the *ccr4Δ* and *ccr4Δ pbp1Δ* mutants (Fig 2C,2D and 2E). We also examined the *LRG1* mRNA level in each of polysome fractions from the *ccr4Δ pbp1Δ pan2Δ* mutant at 72 h time point (Fig 2E). While Lrg1 protein level was decreased in the *ccr4Δ pbp1Δ pan2Δ* mutant at 72 h time point (Fig 4A), *LRG1* mRNA was still enriched at heavy polysome fractions in the *ccr4Δ pbp1Δ pan2Δ* mutant (Fig 2E). Thus, Pbp1 may enhance the translation in the absence of Ccr4 and Pan2 in an independent manner of the association of *LRG1* mRNA to polysomes.

To confirm the involvement of *LRG1* poly(A) tail length in the regulation of *LRG1* mRNA translation, we overexpressed *PAN2* in the *ccr4Δ* mutant and then examined Lrg1 protein level. We have previously reported that *PAN2* overexpression from the multi-copy plasmid suppresses the growth defect of the *ccr4Δ khd1Δ* mutant [14]. *PAN2* overexpression partially decreased *LRG1* poly(A) tail length (Fig 4C, lane 3). However, the overexpression of *PAN2* did not reduce Lrg1 level in the *ccr4Δ* background in the stationary phase (Fig 4B). It may be more Pbp1 loaded on long *LRG1* poly(A) tail and inhibit the access of Pan2. We also examined the expression of Lrg1 protein in the *ccr4*Δ *pan2*Δ double mutant from the log phase to the stationary phase. At 48 h time point, the Lrg1 protein in the *ccr4*Δ *pan2*Δ double mutant was maintained at high level similar to that in *ccr4Δ* mutant (Fig 4D). However, at 72 h and 96 h time points, Lrg1 protein levels in the *ccr4*Δ *pan2*Δ double mutant were decreased compared to those in *ccr4Δ* mutant. The *LRG1* poly(A) tail length in *ccr4Δ pan2Δ* mutant was more longer than that in *ccr4*Δ mutant at 72 h time point (Fig 4C, lane 4), suggesting that the longer poly(A) tail may interfere the translation of *LRG1* mRNA in the late stationary phase. Alternatively, since the *ccr4*Δ *pan2*Δ double mutant shows more severe growth defect than the *ccr4*Δ single mutant, the decreased protein levels may be caused by the growth defect.

### *MCM2*, *MCM4*, *MCM7*, and *ELM1* show the expression pattern similar to that of *LRG1* in the stationary phase

**Fig 5:**
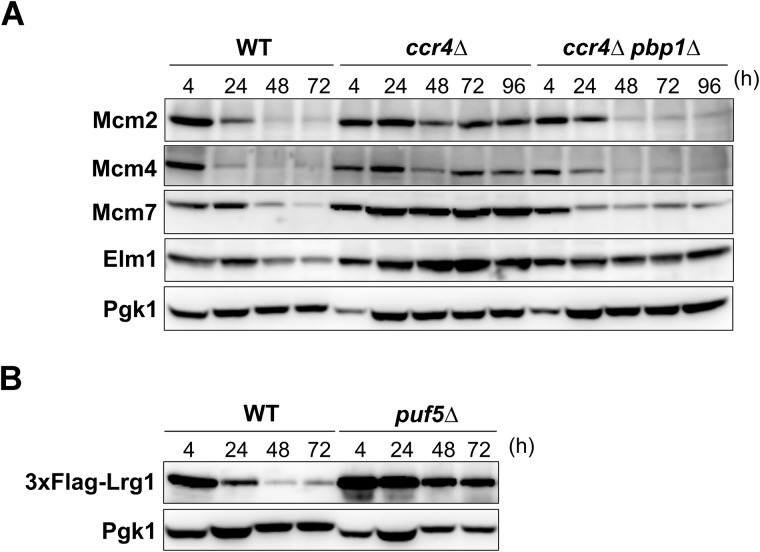
The other target mRNAs of Puf5 including *MCM2*, *MCM4*, *MCM7*, and *ELM1* showed the expression patterns similar to that of *LRG1*. (A) Protein expressions for products of Puf5 target mRNAs in WT, *ccr4Δ*, and *ccr4Δ pbp1Δ* mutant cells. The WT, *ccr4Δ*, and *ccr4Δ pbp1Δ* mutant cells harboring the plasmid pRS314-3FLAG-LRG1 were grown at 28°C from the log phase to the stationary phase in SC-Trp media. The cells were collected at the indicated times, and cell extracts were prepared for immunoblotting with anti-Flag (3xFlag-Lrg1), anti-Mcm2, anti Mcm4, anti-Mcm7, anti-Elm1, and anti-Pgk1 antibodies. Pgk1 was used as the loading control. (B) The Lrg1 protein level in WT, *puf5Δ* mutant in the stationary phase. WT and *puf5Δ* mutant strains harboring the plasmid pRS314-3FLAG-LRG1 were grown at 28°C from the log phase to the stationary phase in SC-Trp media. The cells were collected at the indicated times, and cell extracts were prepared for immunoblotting with anti-Flag (3xFlag-Lrg1) and anti-Pgk1 antibodies. Pgk1 was used as the loading control.

We have previously shown that the suppression of the *ccr4Δ* mutation by the *pbp1Δ* mutation was not identical to that by the *lrg1Δ* mutation [14]. Whereas the *pbp1Δ* mutation suppressed both the slow growth phenotype at room temperature and the growth defect at 37°C of the *ccr4Δ khd1Δ* double mutant, the *lrg1Δ* mutation suppressed only the growth defect at 37°C, but not the slow growth phenotype at room temperature. Thus, deletion of *PBP1* can suppress the growth defect of the *ccr4Δ* mutant by decreasing the expression probably not only of Lrg1 protein but also of other proteins, in the stationary phase. We then searched for the other potential candidates similar to *LRG1* gene. The *LRG1* mRNA is one of the targets of Puf5/Mpt5, an RNA binding protein [28–30]. Puf5 binds to specific site in 3'-UTR of its target mRNAs and recruits Ccr4-Not complex for deadenylation [8, 9]. Among the targets of Puf5 [29, 30], we investigated the protein levels of *MCM2*, *MCM4*, *MCM7*, and *ELM1* genes in WT, *ccr4*Δ, and *ccr4*Δ *pbp1*Δ mutant strains in the longer culture, because the antibodies for these proteins were commercially available. The data showed that the protein levels of these genes were strongly decreased in WT but slightly decreased in the *ccr4*Δ mutant after 48 h of culture (Fig 5A, WT and *ccr4*Δ, 48 h and 72 h). Similar to the results of Lrg1 protein, deletion of *PBP1* also reduced these protein levels in the stationary-phase *ccr4Δ* mutant cells (Fig 5A, *ccr4Δ pbp1Δ*, 48 h and 72 h). The poly(A) tail lengths of these mRNAs were also increased in the *ccr4Δ* mutant and decreased in the *ccr4Δ pbp1Δ* mutant at 48 h of culture (data not shown). These data suggest that Ccr4 is required for translational repression not only of *LRG1* mRNA but also of other Puf5 target mRNAs in the stationary phase. We also addressed to the question whether Puf5 is required for the regulation of *LRG1* mRNA. At 48 h and 72 h of *puf5Δ* mutant culture, Lrg1 protein level was decreased but still remained higher than those in WT cells (Fig 5B), suggesting that Puf5 as well as Ccr4 is required for the down-regulation of Lrg1 in the stationary phase.

## Discussion

### The *LRG1* poly(A) tail length positively correlated to *LRG1* mRNA and protein levels in the stationary phase

The increase in poly(A) tail length is supposed to inhibit mRNA degradation and enhance translation *in vivo* [5, 6, 11]. In contrast, Traven et al. have reported that long poly(A) tails of the mRNAs encoding regulators of septin assembly do not affect their mRNA and protein levels in the *ccr4Δ* mutant [12]. In addition, in a genome-wide analysis, Subtelny et al. have shown that the poly(A) tail length positively correlates to translation efficiency only in early zebrafish and frog embryo, and deadenylation primarily enhances mRNA decay [31]. The poly(A) tail length, however, does not affect translation in yeast [31]. Therefore, it is still ambiguous about the relationship between poly(A) tail length and translational control. In this study, for the first time, we have provided the evidences that poly(A) tail length positively correlates to the level and translational efficiency of *LRG1* mRNA in the stationary phase, but not in the log phase. Consistent with the report of Traven et al. [12], the longer *LRG1* poly(A) tail in the *ccr4Δ* mutant did not affect Lrg1 protein level in the log phase. It is likely that poly(A) tail length is not important to translational control of *LRG1* mRNA in the log phase. The regulation of mRNA stability and translational efficiency in the log phase may involve other factors rather than poly(A) tail. Interestingly, when the cells reached saturated cell density, deletion of *CCR4* has stronger effect on Lrg1 protein level rather than on *LRG1* mRNA level. The aberrant *LRG1* mRNA and protein levels in the *ccr4Δ* mutant were correlated to the long *LRG1* poly(A) tail length, suggesting that the down-regulation of *LRG1* in the stationary phase requires the deadenylation of mRNA that is mediated by Ccr4. The longer poly(A) tail length, where more Pab1 may bind to and facilitate the formation of mRNP loop structure, inhibits mRNA degradation and facilitates the translation, and vice versa. The *pbp1Δ* mutation decreased the *LRG1* poly(A) tail length to the similar extend in WT in the stationary-phase *ccr4Δ* mutant cells, and then decreased the aberrant *LRG1* mRNA and protein levels. Thus, the poly(A) tail length and Ccr4 deadenylase seems to play an important role in regulation of *LRG1* mRNA and protein levels in the stationary phase rather than that in the log phase.

Since deletion of *PBP1* reduced the *LRG1* poly(A) tail length in the stationary-phase *ccr4Δ* mutant, it comes to the question how Pbp1 contributes to the regulation of *LRG1* poly(A) tail? Mangus et al. have shown that Pbp1 negatively regulates Pan2 deadenylase by disturbing the Pab1-Pan2 interaction, and that the cell extract from *pbp1Δ* single mutant in the stationary phase has stronger deadenylase activity than that in WT *in vitro* [20, 21]. Consistently, we found that the shortening of the *LRG1* poly(A) tail length in the *ccr4Δ pbp1Δ* mutant required Pan2 deadenylase *in vivo*, and that Pbp1 inhibited Pan2 activity only in the stationary phase but not in the log phase. It is thought that the Pan2-Pan3 complex act as primary deadenylase [32]; however, here we found that this complex could also act as secondary cytoplasmic deadenylase in the absence of both Ccr4 and Pbp1 in the stationary phase. Although *LRG1* mRNA harbored longer poly(A) tail in the stationary-phase *ccr4Δ pbp1Δ pan2Δ* triple mutant cells, Lrg1 protein level was not increased in the cells, suggesting that the translation of *LRG1* mRNA still requires Pbp1. On the other hand, overexpression of *PAN2* had little effect on *LRG1* poly(A) tail length and did not reduce Lrg1 protein level in the *ccr4Δ* mutant. It may be explained by the unusual Pbp1 loading onto long *LRG1* poly(A) tail, resulted in blocking of the Pan2 access to the *LRG1* poly(A) tail. Taken together, we first described here that Pbp1 together with the Pan2-Pan3 complex contributes to the regulation of poly(A) tail length in the stationary phase *in vivo* through a particular example, *LRG1* poly(A) tail. Further analysis should be needed to elucidate the physiological role of Pan2 inhibition by Pbp1 in the stationary phase.

### Ccr4 is required not only for translational repression of *LRG1* mRNA but also for global translational repression in the stationary phase

The yeast cells enter into the stationary phase when the carbon source is depleted in the media. To adapt to this environmental signal, cells reduce cellular activities including protein synthesis and other metabolic processes to save energy for long-term survival and turn into quiescent state [27, 33]. There are several reports that translational repression required the mRNA regulatory factors upon nutrient depletion. For example, Coller et al. have shown that the decapping activators Dhh1 and Pat1 are required for general translational repression in the glucose starvation condition [34]. In addition, Preissler et al. have revealed that Not4, a component of Ccr4-Not complex, is also required for translational repression in response to nutrient withdrawal [25]. In this study, we have shown that the translation of *LRG1* mRNA is repressed prior to the decrease in *LRG1* mRNA level upon the stationary phase, and this translational repression requires the Ccr4 deadenylase. Intriguingly, the active translating ribosomes were decreased in the stationary-phase WT cells but still remained abundant in the stationary-phase *ccr4Δ* mutant cells, suggesting that Ccr4 is required not only for translational repression of *LRG1* mRNA but also for global translational repression. Taken together with previous observations [25, 34], translational repression is tightly coupled with mRNA decay, and requires mRNA degradation machinery such as the Ccr4-Not complex and the decapping activators.

How does Ccr4 repress the global translation in the stationary phase? One of the possibilities is that Ccr4 shortens the poly(A) tail length in order to decrease mRNA stability and translation efficiency through disrupting mRNP loop structure. The mRNAs harboring shortened poly(A) tail would avoid the aberrant translations. In case of the *LRG1* mRNA, the *pbp1Δ* mutation suppressed the longer poly(A) tail caused by the *ccr4Δ* mutation, and then reduced the *LRG1* mRNA and Lrg1 protein levels in the stationary phase. However, the *pbp1Δ* mutation did not suppress the aberrant translating polysomes of the stationary-phase *ccr4Δ* mutant cells. Thus, Pbp1 may regulate the translation in a gene specific manner rather than a general consequence through the interaction with ribosomes. Since Caf1, a deadenylase catalytic component of Ccr4-Not complex, has been reported to repress the translation independent of its deadenylation in *Xenopus laevis* oocytes [26], Ccr4 may have a translational repression function independent of its deadenylase activity. However, the deadenylase-dead *CCR4 (D713A)* mutant could not decrease high Lrg1 protein level in the stationary-phase *ccr4Δ* mutant cell, suggesting that translation repression role of Ccr4 required its deadenylase activity. As to the regulation of translational repression by Ccr4 in the stationary phase, there are several lines of evidence that support the relationship between Ccr4 and protein kinase A (PKA) pathway. PKA pathway is known to be inactivated in the stationary phase. Lenssen et al. suggested that Ccr4 acts as downstream activator of PKA pathway in the regulation of Msn2/Msn4 dependent transcription [35, 36]. However, translational activity was still abundant in the absence of Ccr4 in the stationary phase, and constitutively activated PKA pathway also maintained high Lrg1 protein level (data not shown), implicating that PKA pathway might be the downstream effector of Ccr4 instead. Perhaps the defect in the inactivation of PKA activity in the stationary-phase *ccr4Δ* mutant cells could cause high translational activity, and further analysis need to be done to clarify this involvement. Taken together, we found here that Ccr4 deadenylase is required for global translational repression including translational repression of *LRG1* mRNA in the stationary phase.

### Puf5 contributes to the down-regulation of its target mRNAs in the stationary phase

Beside *LRG1* mRNA, we have also found that the other target mRNAs of Puf5 including *MCM2*, *MCM4*, *MCM7*, and *ELM1* are also up-regulated in a manner dependent on Pbp1 in the stationary-phase *ccr4Δ* mutant cells. Previous report showed that Pbp1 also affects the translation of *HO* mRNA [18], another target of Puf5, raising the possibility of the involvement of Pbp1 specifically in the translational regulation of Puf5 target mRNAs. Recent finding revealed that ataxin-2, the human ortholog of Pbp1, stabilizes mRNAs by binding to specific site within 3'-UTR and enhance translation [37]. Likewise, the 3'-UTR of Puf5 target mRNAs may contain the specific binding site where Pbp1 binds to and ensures the translation. Moreover, the longer poly(A) tail found in the *ccr4Δ* mutant would provide the opportunity for the binding of numerous Pbp1 to the specific sites and facilitate the translation. On the other hand, Puf5 recruits Ccr4-Not complex for deadenylation by binding to the specific site in the 3'-UTR of its target mRNAs [8, 9]. We have also found that Lrg1 protein level in the *puf5Δ* mutant is higher than that in WT in the stationary phase, indicating that Puf5 contributes to the down regulation of its target mRNAs in the stationary phase. Furthermore, Puf5 contains phosphorylation motif of PKA [38], implicating the possibility that Puf5 would become more active and would repress the translation of their target mRNAs, together with Ccr4, in the stationary phase, when the PKA activity is very low.

In summary, the results presented in this study demonstrate that *LRG1* poly(A) tail length is important to *LRG1* mRNA and protein levels in the stationary phase. Although the role of poly(A) tail has been discussed in a number of studies, we identified here the first evidence in which poly(A) tail length positively correlates with translational efficiency in the stationary phase in yeast. In addition, we found that global translational repression that happens in the stationary phase requires Ccr4 deadenylase. It is likely that Ccr4 plays an important role in proper cellular homeostasis upon the stationary phase by inhibiting aberrant translation of Puf5 target mRNAs which is facilitated by Pbp1. Furthermore, we found that Pbp1 together with the Pan2-Pan3 complex regulates *LRG1* poly(A) tail *in vivo*. Further works need to be carried out to provide valuable insights into the molecular mechanism of translational repression by cytoplasmic deadenylase Ccr4 in the stationary phase.

## Materials and methods

### Strains and media

*Escherichia coli* DH5α strain was used for DNA manipulations. The yeast strains used in this study are isogenic derivatives of the W303 background and are listed in S1 Table. The deletion mutants were generated by a PCR-based method, as described previously [39], and were verified by PCR to confirm complete deletion at the expected locus. Yeast strains were manipulated according to standard procedures [40]. The media used in this study including rich medium (YPD) and synthetic complete medium (SC). SC media lacking amino acids or other nutrients (e.g. SC-Trp corresponding to SC lacking tryptophan) were used to select the transformants. The glucose level in the media was measured by using the Glucose (GO) Assay Kit (Sigma), and ethanol level was measured by using the Ethanol Assay Kit (DIET-500) (BioAssay Systems).

### Plasmids

Plasmids used in this study are listed in S2 Table. The pRS314-3FLAG-LRG1 plasmid was constructed as follow. The fragment encoding *LRG1* promoter and the fragment encoding *LRG1* ORF—*LRG1* terminator were obtained by PCR from genomic DNA using two pairs of primers (CTAAAGGGAACAAAAGCTGGGTACCTATGGGCAAACAATATAACCC and GATAACCAGCAGAATTTTGAACCATGGCTCACCTCCGGTACTTGT; ACAAGTACCGGAGGTGAGCCATGGTTCAAAATTCTGCTGGTTATC and CTCACTATAGGGCGAATTGGAGCTCATATTCAATGGTGTCATTAAT) to introduce an additional *Nco*I site right after the start codon. Two fragments were inserted into between *Kpn*I and *Sac*I sites of the pRS314 plasmid using gap repair cloning [41]. The synthetic fragment encoding 3xFLAG with two flanking *Nco*I sites (5'-CATGGACTACAAAGACCATGACGGTGATTATAAAGATCATGACATCGATTACAAGGATGACGATGACAAGGG-3' and 3'-CTGATGTTTCTGGTACTGCCACTAATATTTCTAGTACTGTAGCTAATGTTCCTACTGCTACTGTTCCCGTAC-5') was then annealed and inserted into the N-terminal of *LRG1* ORF. The plasmid YEplac195-LRG1 and YEplac195-PAN2 were used to over-express *LRG1* and *PAN2* genes, respectively. The plasmids YCplac33-CCR4 and YCplac33-CCR4-D713A express the wild-type *CCR4* allele and the deadenylase-dead *CCR4 (D713A)* allele [24], respectively. The plasmids pCgLEU2, pCgHIS3, and pCgTRP1 are pUC19 carrying the *Candida glabrata LEU2*, *HIS3*, and *TRP1* genes respectively, were used for gene deletion experiments [42].

### RNA extraction, qRT-PCR, and poly(A) tail length assay

Cells were grown from the exponential phase to the stationary phase in YPD medium or SC-Trp medium and then harvested at the indicated times. Total RNAs were then prepared using ISOGEN reagent (Nippon Gene) and the RNeasy Mini kit (Qiagen). First strand of cDNAs were generated using the Prime Script RT reagent Kit (Takara). The cDNAs were quantified by a quantitative real-time RT-PCR (qRT-PCR) method using a 7500 fast real-time RT-PCR system (Applied Biosystems) with SYBR Premix Ex Taq (Takara). The *LRG1* primers (ACCTGCCAAGACTGTCAGAAAC and TAATCCACGCAATGGGGTATC) and *SCR1* primers (AACCGTCTTTCCTCCGTCGTAA and CTACCTTGCCGCACCAGACA) [43] were used to analyze the mRNA levels of *LRG1* and *SCR1*. The fold changes in mRNA levels were calculated by using the delta delta Ct method and normalized to the *SCR1* reference gene. The statistical analysis was performed with Excel (Microsoft) using Tukey’s test, and differences were considered significant when p < 0.05. The poly(A) tail length of *LRG1* mRNA was measured by using the poly(A) tail length assay kit (Affymetrix) according to the manufacturer's instruction. A fragment including *LRG1* poly(A) tail was amplified by using the forward primer anneals to *LRG1* 3'-UTR (CCAGTATGCTATGGAAATGG) and the universal reverse primers included in the kit. The average length of poly(A) tail were determined by sequencing.

### Protein extraction, western blot analysis, and antibodies

The cells collected from indicated times were then treated with sodium hydroxide for protein extraction, as described previously [44]. Protein samples were loaded on to an 8% or 10% SDS-PAGE gel for protein electrophoresis and then transferred to a PDVF membrane (Millipore) for Western blot analysis. Anti-FLAG polyclonal antibody M2 (Sigma), anti-Mcm2 polyclonal antibody N-19 (Santa Cruz), anti-Mcm4 polyclonal antibody yC-19 (Santa Cruz), anti-Mcm7 polyclonal antibody yN-19 (Santa Cruz), and anti-Elm1 polyclonal antibody y-640 (Santa Cruz) were used to detect 3Flag-Lrg1, Mcm2, Mcm4, Mcm7, and Elm1, respectively. The monoclonal anti-Pgk1 antibody 22C5D8 (Invitrogen) was used to detect Pgk1, as the loading control, since Pgk1 is reported to be a very stable protein based on its half-life [45]. Detection was carried out by using a LAS-4000 (Fuji Film) with Immobilon Western (Merck Millipore). Signal intensities were quantified by means of Image Quant (GE Healthcare).

### Polysome analysis

Cycloheximide was added to the cultures to the final concentration 100 μg/ml, and agitated for 15 min to stop the translation. The cells were harvested and resuspended in 0.5 ml lysis buffer (10 mM Tris-HCl [pH 7.5], 100 mM NaCl, 30 mM MgCl_2_, 100 μg/ml cycloheximide, 200 μg/ml heparin, 0.1% dithiothreitol, 10 μg/ml aprotinin, 10 μg/ml leupeptin) and then mixed with 0.5 ml glass beads. The cells were lysed by bead beating 4 times, each time for 30 s with 30 s interval on ice. After bead beating, 0.5 ml lysis buffer was added, and centrifuged at 14,000 rpm for 10 min at 4°C to collect the supernatant. Twenty A260 nm units of the supernatant were loaded on top of sucrose gradients (10% – 50% w/v). Polysomes were fractionated by centrifugation at 27,000 rpm for 3 h at 4°C with a SW28 Ti rotor (Beckman Coulter). The gradient was continuously collected from the Gradient Station (Biocomp), and the collection line was connected to a UV detector to monitor the 254 nm absorbance. Sixteen fractions (1.9 ml/fraction) were collected by a fraction collector. The RNA from polysomes fractions were precipitated by ethanol overnight at -30°C and then purified by using RNeasy Mini kit (Qiagen). The cDNAs were generated from the same volume RNA samples using the Prime Script RT reagent Kit (Takara). The *LRG1* cDNA was amplified by Blend Taq (Toyobo) with specific primers (TCTCGATGATAAGGGCTATCAG and TAACACGCTGTTTCTCATCCTC).

## Supporting information

S1 Table Strains used in this study. (DOCX)

S2 Table Plasmids used in this study. (DOCX)

S1 File References. (DOCX)
