# Supplementary material for: Cytoplasmic deadenylase Ccr4 is required for translational repression of LRG1 mRNA in the stationary phase
Source: PLoS One. 2017 Feb 23;12(2):e0172476. doi: 10.1371/journal.pone.0172476 (PMC5322899; doi:10.1371/journal.pone.0172476)
Supplement: S1 Table — (DOCX) [file pone.0172476.s001.docx]

S1 Table. Strains used in this study

| Strains | Genotype | Reference |
| --- | --- | --- |
| 10B | *MATα ade2 trp1 can1 leu2 his3 ura3 GAL psi+ HOp-ADE2-HO 3' UTR* | [46] |
| 10BD | *MATa/MATα ade2/ade2 trp1/trp1 can1/can1 leu2/leu2 his3/his3 ura3/ura3* | [46] |
| *ccr4∆* | *MATa ade2 trp1 can1 leu2 his3 ura3 ccr4Δ::CgLEU2* | [13] |
| *ccr4∆ pbp1∆* | *MATa ade2 trp1 can1 leu2 his3 ura3 ccr4Δ::CgLEU2 pbp1Δ::CgHIS3* | [14] |
| *ccr4∆ pbp1∆ pan2∆* | *MATa ade2 trp1 can1 leu2 his3 ura3 ccr4Δ::CgLEU2 pbp1Δ::CgURA3 pan2Δ::CgHIS3* | [14] |
| *puf5∆* | *MATa ade2 trp1 can1 leu2 his3 ura3 puf5Δ::CgHIS3* | [47] |
