# Supplementary material for: Cytoplasmic deadenylase Ccr4 is required for translational repression of LRG1 mRNA in the stationary phase
Source: PLoS One. 2017 Feb 23;12(2):e0172476. doi: 10.1371/journal.pone.0172476 (PMC5322899; doi:10.1371/journal.pone.0172476)
Supplement: S2 Table — (DOCX) [file pone.0172476.s002.docx]

S2 Table. Plasmids used in this study

| Plasmids | Relevant markers | Reference |
| --- | --- | --- |
| pRS314 | *TRP1, CEN-ARS* | [48] |
| YEplac195 | *URA3, 2µ* | [49] |
| YCplac33 | *URA3, CEN-ARS* | [49] |
| pRS314-3FLAG-LRG1 | *TRP1, CEN-ARS, pLRG1-3FLAG-LRG1-LRG1 3'-UTR* | This study |
| YEplac195-LRG1 | *URA3, 2µ, pLRG1-LRG-LRG1 3'-UTR* | This study |
| YEplac195-PAN2 | *URA3, 2µ, pPAN2-PAN2-PAN2 3'-UTR* | [14] |
| YCplac33-CCR4 | *URA3, CEN-ARS, pCCR4-CCR4-CCR4 3'-UTR* | This study |
| YCplac33-CCR4-D713A | *URA3, CEN-ARS, pCCR4-CCR4-D713A-CCR4 3'-UTR* | This study |
| pCgLEU2 | *C. glabrata LEU2* in pUC19 | [42] |
| pCgHIS3 | *C. glabrata HIS3* in pUC19 | [42] |
| pCgTRP1 | *C. glabrata TRP1* in pUC19 | [42] |
