# Supplementary material for: Cytoplasmic deadenylase Ccr4 is required for translational repression of LRG1 mRNA in the stationary phase
Source: PLoS One. 2017 Feb 23;12(2):e0172476. doi: 10.1371/journal.pone.0172476 (PMC5322899; doi:10.1371/journal.pone.0172476)
Supplement: S1 File — (DOCX) [file pone.0172476.s003.docx]

**Supporting Information**

**References**

46. Irie K, Tadauchi T, Takizawa PA, Vale RD, Matsumoto K, Herskowitz I. The Khd1 protein, which has three KH RNA-binding motifs, is required for proper localization of ASH1 mRNA in yeast. EMBO J. 2002; 21(5): 1158-67. doi: 10.1093/emboj/21.5.1158 PMID: 11867544

47. Tadauchi T, Matsumoto K, Herskowitz I, Irie K. Post-transcriptional regulation through the HO 3'-UTR by Mpt5, a yeast homolog of Pumilio and FBF. EMBO J. 2001 Feb 1;20(3):552-61. PMID: 11157761

48. Sikorski RS, Hieter P. A system of shuttle vectors and yeast host strains designed for efficient manipulation of DNA in Saccharomyces cerevisiae. Genetics. 1989; 122(1): 19-27. PMID: 2659436

49. Gietz RD, Sugino A. New yeast-Escherichia coli shuttle vectors constructed with in vitro mutagenized yeast genes lacking six-base pair restriction sites. Gene. 1988; 74(2): 527-34. PMID: 3073106
